# Preparation, Physical-Chemical Characterization, and Cytocompatibility of Polymeric Calcium Phosphate Cements

**DOI:** 10.1155/2011/467641

**Published:** 2011-09-20

**Authors:** Rania M. Khashaba, Mervet Moussa, Christopher Koch, Arthur R. Jurgensen, David M. Missimer, Ronny L. Rutherford, Norman B. Chutkan, James L. Borke

**Affiliations:** ^1^Department Oral Biology, Medical College of Georgia, Augusta, GA 30912-1129, USA; ^2^Department Orthopaedic Surgery, Section of Biomaterials, Medical College of Georgia, Augusta, GA 30912-1129, USA; ^3^Department of Dental Materials, Misr International University (MIU), Cairo 11787, Egypt; ^4^Department of Oral Pathology, Cairo University, Cairo 11559, Egypt; ^5^Department of Oral Pathology, Misr International University (MIU), Cairo 11787, Egypt; ^6^Savannah River National Laboratory, Savannah River Nuclear Solutions, Aiken, SC 29808, USA

## Abstract

*Aim*. Physicochemical mechanical and *in vitro* biological properties of novel formulations of polymeric calcium phosphate cements (CPCs) were investigated. *Methods*. Monocalcium phosphate, calcium oxide, and synthetic hydroxyapatite were combined with either modified polyacrylic acid, light activated polyalkenoic acid, or polymethyl vinyl ether maleic acid to obtain Types I, II, and III CPCs. Setting time, compressive and diametral strength of CPCs was compared with zinc polycarboxylate cement (control). Specimens were characterized using X-ray diffraction, scanning electron microscopy, and infrared spectroscopy. *In vitro* cytotoxicity of CPCs and control was assessed. *Results*. X-ray diffraction analysis showed hydroxyapatite, monetite, and brushite. Acid-base reaction was confirmed by the appearance of stretching peaks in IR spectra of set cements. SEM revealed rod-like crystals and platy crystals. Setting time of cements was 5–12 min. Type III showed significantly higher strength values compared to control. Type III yielded high biocompatibility. *Conclusions*. Type III CPCs show promise for dental applications.

## 1. Introduction

The rapidly evolving field of materials science is providing dentistry with new treatments and alternatives. Calcium phosphate materials have been increasingly employed in orthopedic and dental applications. Recently, much attention has been paid to calcium phosphate cements (CPCs) because of their advantages in comparison with calcium phosphate bioceramics, regarding *in situ* handling and shaping abilities [1, 2]. The CPCs are obtained by an acid-base reaction in water between an acidic calcium phosphate and a basic calcium phosphate, giving an intermediate basicity calcium phosphate that precipitates [3]. The use of CPCs with their biocompatibility, potential for osteoconduction and sealing ability, may improve the prognosis of dental treatment. In this regard, CPCs have been explored for the treatment of furcal exposures [4], root sensitivity, open-root apices, and endodontic obturation [5, 6]. The self-setting and biocompatibility properties of CPCs suggest that they would be superior to pure calcium hydroxide. Thus, these materials may have utility for dentine regeneration, pulp capping, and cavity lining [7, 8]. In addition, instead of pure CPC, composites fabricated from calcium phosphates and polymers may have applications as pulp capping and cavity-basing materials [9]. Finally, the release of ions (OH^−^, PO_4_^−3^, and Ca^+2^) from cements with a Ca/P ratio of 1.67 has been reported to have antimicrobial properties. Despite these potential advantages, the usefulness of water-based CPC pastes are limited, since under ambient conditions, they are not highly cohesive and vulnerable to wash out until hardening occurs [10]. Therefore, modifying additives like calcium chloride and sodium alginate were tried as reinforcing materials [11]. In studies on CPCs, sodium alginate or cellulose derivatives dissolved in the cement liquid was suggested for improving the cohesion of CPC pastes. CPCs exhibit relatively low mechanical strength, mechanical properties are relatively weak compared to enamel, dentin, resin-based composites and some dental cements [12]. 

Moreover, in the present state, CPCs do not compare favorably with currently available dental cements in terms of setting time. Therefore, modification of powder composition and properties, introduction of reinforcing materials into liquids, and/or changing the mixing methods are necessary for improvements of CPCs to meet the requirements for dental applications, and thus replace calcium hydroxide pastes currently used in dentistry with more durable materials bearing setting time (2–8) minutes and a high compressive strength (50–80 MPa) while retaining their biological advantages. 

 In this study, we tested the hypothesis that incorporation of several polymeric acids into traditional CPCs would produce formulations with promising physical, mechanical and biological properties to permit wide dental applications. The present study tests this hypothesis by measuring: (1) the physical chemical characteristics of three novel CPC formulations derived from a mixture of CPC powder with three aqueous solutions of polymeric acids (modified polyacrylic acid, light-activated modified polyalkenoic acid, and 35% w/w polymethyl vinyl ether maleic acid as compared to a clinically available zinc polycarboxylate cement, (2) the compressive and diametral tensile strength of these polymeric cements, and (3) using the direct contact cell culture format, we compare the cytotoxic properties of these CPC formulations and one clinically available zinc polycarboxylate cement. (The biocompatibility examined on human gingival fibroblast cells and the cell viability quantified using MTT assay.)

## 2. Materials and Methods

Three polymeric calcium phosphate cements and zinc polycarboxylate cement were evaluated (Table 1). The powder component of the calcium phosphate cements was derived from a mixture of monocalcium phosphate monohydrate (MCPM), calcium oxide (CaO), and synthetic hydroxyapatite (SHAp6). Three types of aqueous solutions of polymeric acids were used for mixing these powders to obtain novel polymeric-CPC formulations.

### 2.1. Preparation of Calcium Phosphate Cement Powder

Both the monocalcium phosphate monohydrate (MCPM) and the calcium oxide (CaO) powder were crushed separately in an agate mortar and then sieved to obtain an average particle size of 80 *μ*m 0.074 mm sieve was used (mesh size no. 200). A mixture of MCPM and CaO was prepared at a Ca/P ratio of 1 : 67 (same ratio present in bone and dentin) [13]. This mixture constituted 60% of the total weight of the powder. Synthetic hydroxyapatite (SHAp6) was chemically precipitated using appropriate amount of Ca(OH)_2_ and concentrated phosphoric acid to maintain Ca/P ratio of 1.67. Synthetic hydroxyapatite (SHAp6) (40 wt %) was incorporated to increase the ultimate strength of the cement and to act as seeds for nucleation of more hydroxyapatite [14, 15]. The powder was mechanically mixed in a Turbula shaking apparatus (Willy Bachofen, Basel, Switzerland).

### 2.2. Preparation of Aqueous Solution of Polymeric Liquids

Three types of polymeric liquids (Table 1) were mixed individually with the calcium phosphate powder: (1) modified polyacrylic acid of zinc polycarboxylate cement (Type I cement), (2) visible light-cured modified-polyalkenoic acid of glass ionomer as supplied by manufacturer (Type II cement), and (3) a 35% (w/w) aqueous solution of polymethyl vinyl ether maleic acid, prepared by dissolving 35 g of the white powder of polymethyl vinyl ether maleic anhydride (PMVE-MA) copolymer (molecular weight 50,000) in 100 mL of distilled water. The 35% w/w aqueous solution of PMVE-Ma was mixed with CPC powder to form the polymeric-CPC cement (Type III cement).

### 2.3. Preparation of Polymeric CPC Cements

Several pilot studies were performed to select the best powder to liquid ratio (4 : 1) that produced good handling characteristics and working time. Zinc polycarboxylate cement was mixed according to the manufacturer's instructions and considered as an additional control group. These newly formulated calcium phosphate cements composed of one form of powder and three different types of liquid were evaluated and compared to the control group (zinc polycarboxylate) cement in regards to the following.

### 2.4. Determination of Setting Time

 The initial setting time of each of the cement mixtures under investigation was determined according to the method described in the ANSI/ASTM-C-191-1977 [16, 17] using Gillmore needle (Zur nadel-Ton Industries, Berlin, Germany) (113.4 gm. and diameter 2.13 mm). The initial setting time was determined as the time elapsed from the start of mixing until the needle fails to leave an indentation deeper than 1 mm on the cement surface. The setting time was recorded to the nearest minute and the test was repeated five times. The mean and standard deviation for both the experimental and the commercial zinc polycarboxylate cement were calculated.

### 2.5. Assessment of the Mechanical Properties of the Prepared Cements

**(a) Preparation of the Compressive Strength Test Specimens**

A total of 120 cylindrical specimens of 6mm diameter and 12 mm height were prepared according to the ISO specification no. 4104 [18] for zinc polycarboxylate cement. The cement pastes were mixed as previously described and inserted into a split metal mold. For the visible light cured version of polymeric CPC, special Teflon mould was used, and the material was built up in small increments. Each increment was light cured for 60 s from each side with a composite curing unit with an output of at least 500 MW cm^−2^ (Kulzer Translux CL, Wehrheim,Germany).

The split molds were covered with glass plates, and specimens were kept undisturbed for 60 minutes at 37°C under 100% relative humidity before separation from the mould [19]. The specimens were immersed in distilled water for 1 hour, 24 hr, 1 week, 4 weeks, and 8 weeks before the compressive strengths were measured. Each specimen was placed in 5 mL of distilled water. The distilled water was renewed every week.

**(b) Preparation of the Diametral Tensile Strength Specimens**

For the diametral tensile strength test, 120 disc specimens of 6 mm diameter and 3 mm height were prepared for each type of cement [20]. The specimens were prepared as previously described for the compressive strength test.

**(c) Testing Procedure**

The compressive and diametral tensile strength test of each type of cement were determined after 1 hour, 24 hours, 1 week, 4 weeks, and 8 weeks storage in distilled water using a Universal Testing Machine (Com-Ten Industries, Inc., Fla, USA) at a cross-head speed of 0.5 mm/min. The compressive strength was measured by dividing the maximum load in compression on the ends of the cylindrical specimens by the original cross-sectional area of the test specimen [21]. While the diametral tensile strength (DTS) was calculated according to the equation (DTS = 2*P*/*πDT*), where *P* is the applied load, *D* is the diameter of the cylinder, and *T* is the thickness of the specimen. A sheet of filter paper (Whatman Type no. 1, Whatman international, Springfield Mill, Maidstone, Kent, England) was placed underneath, and another sheet was placed on the top of the cement specimen during the loading.

All data recorded were subjected to one-way analysis of variance (ANOVA) followed by (where appropriate) the Tukey-*B* test to determine the level of significance between the experimental groups.

### 2.6. Infrared Spectroscopy Analysis (IR)

Fragments of the set cements used for the compressive strength test (24 hours after mixing) were ground and weighed to obtain 1 gram of each set material and were mixed with a measured amount of potassium bromide in an agate mortar. After pressing the mixtures into rigid pellets, IR analysis was performed using a (Perkin Elmer IR spectrometer, Model 1403, USA) at a wavelength range between 4000 and 600 cm^−1^ [19, 20]. The three types of polymeric liquids were also analyzed by IR spectroscopy to obtain a base line reading.

### 2.7. Compositional Analysis

The CPC powder components before and after mixing with the liquid and setting were manually ground to a fine powder in an agate mortar for X-ray diffraction analysis (XRD) analysis. The XRD patterns were collected on a theta-theta PANalytical X'Pert Pro X-ray diffractometer. The instrument was scanned over a 5–70° 2*θ* range with a 0.0167° step size and a dwell time of 99.695 s/2.122° (2*θ*) for a total measurement time of ~1 hr. A Ni-filter was used instead of a graphite monochromator to block the *k*ß radiation. The instrument was run at 45 kV and 40 mA. 

Compound search-match identification was performed with jade soft ware (Version 9) from Materials Data Inc. using the latest inorganic PDF4 powder diffraction data base from the International Centre for Diffraction Data (ICDD).

### 2.8. Scanning Electron Microscopy

 The evolution in morphology of the crystalline structures formed during the process of cement setting (24 hours after mixing) was observed by examining the longitudinal and the fractured surface of the samples using scanning electron microscopy (SEM) (LEO 1450VP, Carl Zeiss SMT, Oberkochen, Germany).

### 2.9. Cell Culture Experiments

Sample preparation was performed aseptically to prevent the risk of biological contamination during the cytotoxicity testing [22]. Zinc polycarboxylate cement was prepared according to the manufacturer's instructions. 

Six discs for eachcement (Types I, II, and III CPCs and zinc polycarboxylate cement) were fabricated in sterile Teflon molds 5.5 mm in diameter and 3 mm thick. The materials were packed into the mold and allowed to set at room temperature (25°C) before testing. Teflon discs were used as a negative control.

### 2.10. In Vitro Biological Testing

Specimens (*n* = 6) were tested for *in vitro* cytotoxicity by placing them in direct contact format (ISO10993) [23] using human gingival fibroblast cells (HGFs) obtained from the Medical College of Georgia School of Dentistry Clinics after obtaining approval from the Medical College of Georgia Human Assurance Committee. Primary cultures of HGFs were established from healthy (noninflamed) tissue removed during routine surgical procedures, using a slight modification of a previously established protocol. Procedural details for this method have been published elsewhere [24, 25]. Twenty-four hours prior to the addition of the specimens, the cells were plated at 4 × 10^4^/well in a 24-well format in 1 mL of medium per well, then specimens were immediately (<1 min) added to the center of each well and secured such that the sample could not move. The ratio of the surface area of the discs to the volume of medium was within the range of 1.2 mm^2^/mL as recommended by the International Standards Organization. The cells and specimens were incubated at 37°C for 72 hours in 5% CO_2_, 95% air to allow attachment of the fibroblasts to the bottom of the wells. After this interval, the specimens were removed from cell culture, rinsed twice with 18 M ohm sterile water, and stored in sterile phosphate buffered saline. The entire set of experiments was repeated to assess the reproducibility of the assay as applied to these materials. The aging times were selected to extend intervals used and to obtain an indication of the trend of cytotoxicity over time.

Cellular activity was assessed by measuring mitochondrial succinic dehydrogenase (SDH) activity via the MTT colorimetric assay [26] after 1, 2, 3, and 4 weeks. Specimens were removed from each well, and the remaining cells were washed carefully with 1.0 mL of phosphate-buffered saline (pH 7.4). A 1 mg/mL MTT solution [3-(4,5-dimethyl–thiazol-2-yl-)-2,5-diphenyl tetrazolium bromide-succinate] was added for 45 min at 37°C, after which the reaction was quenched with the addition of 0.5 mL of 4% Tris-formalin (pH 7.4) for 2-3 min. The MTT-formalin solution was removed, the cell monolayer was allowed to dry for 5–10 min then washed in 1.0 mL of water, and any MTT-formazan formed by SDH activity in the cells was solubilized with 6% dimethylsulfoxide-NaOH (0.1 N NaOH in DMSO). An aliquot of the resulting solution was transferred to a 96-well flat-bottomed tray, and the optical density was measured at 562 nm, the absorption peak of the formazan. Six replicates of each extract or control were performed in each test. Cytotoxicity was expressed as a percentage of the Teflon negative control.

### 2.11. Statistical Analysis

For mitochondrial activity, the means and standard deviations of the MTT-formazan optical densities to Teflon controls were calculated. Statistical difference between the calcium phosphates and the controls was determined using analysis of variance (ANOVA) with Tukey *post hoc* multiple comparison intervals (*α* = 0.05). 

## 3. Results

### 3.1. Setting Time

The results of the initial setting time are presented in (Table 2). The handling properties of polymeric calcium phosphate cements were acceptable from the stand point of working time.

### 3.2. Mechanical Properties

**Figure 1:**
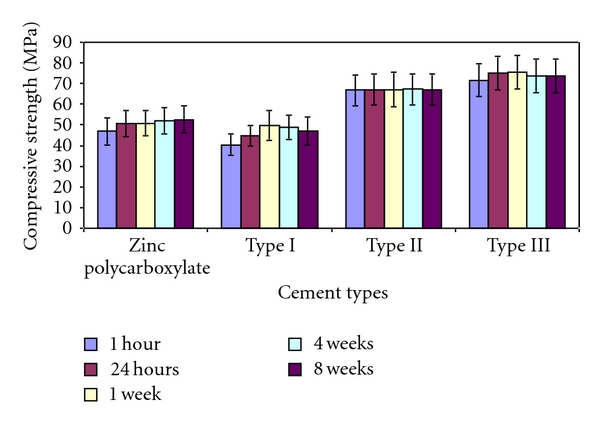
Histogram showing the mean compressive strength of zinc polycarboxylate cement and the three polymeric CPCs in MPa.

**Figure 2:**
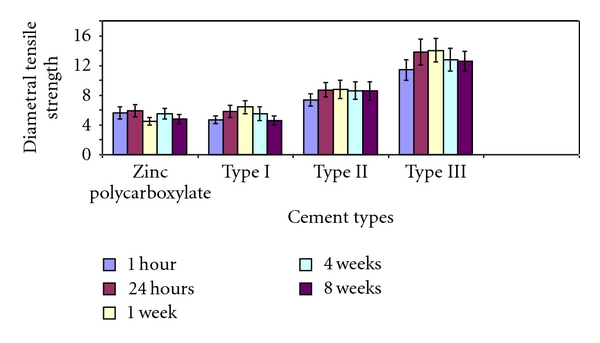
Histogram showing the mean diametral strength of zinc polycarboxylate cement and the three polymeric CPCs in MPa.

The mean values of compressive strength and diametral tensile strength of zinc polycarboxylate cement (control group) and the three polymeric calcium phosphate cements (Types I, II, and III) are listed in (Tables 3 and 4) and illustrated graphically in (Figures 1 and 2).

**(a) Compressive Strength**

At all the storage periods, from 1 hr to 8 weeks, the compressive strength values of polymeric calcium phosphate cement (Type III) derived from 35% w/w aqueous solution of PMVE-Ma were higher than that of zinc polycarboxylate cement (control) and the other two polymeric calcium phosphate cements (*P* < 0.05). The mean compressive strength values of visible light cured (VLC) polymeric CPC cement (Type II) were also higher than that of zinc polycarboxylate cement (*P* < 0.05). Type I polymeric CPC showed the least compressive strength values. When comparing the effect of storage time on the compressive strength of the individual cements, the compressive strength of zinc polycarboxylate, significantly increased from 1 hour (46.85 MPa) to 1 week (50.87 MPa), then significantly increased up to 8 weeks (52.60 MPa). The mean value of compressive strength of (Type I) polymeric CPC significantly increased from 1 hour (40.42 MPa) to 4 weeks (48.74 MPa) then slightly decreased at the end of the eighth week (46.91 MPa).

The mean value of compressive strength of (Type II) derived from visible light cured (VLC) polyalkenoic acid slightly increased from 1 hour (66.86 MPa) to one week (67.15 MPa) and remained nearly constant to the end of the eighth week (67.12 MPa).

The mean value of compressive strength of (Type III) polymeric, CPC derived from (PMVE-Ma), significantly increased from 1 hour (71.68 MPa) up to one week (75.56 MPa) then slightly decreased at the end of the eighth week (73.59 MPa).

**(b) Diametral Tensile Strength**

At all the storage periods from 1 hour to 8 weeks, the mean diametral strength values of polymeric calcium phosphate cement (Type III) and Type (II) were higher than that of zinc polycarboxylate cement and (Type I) polymeric calcium phosphate cement (*P* < 0.05). When comparing the effect of storage time on the mean values of diametral tensile strength, zinc polycarboxylate cement showed the highest diametral strength values at the end of the first week (4.49 MPa) then gradually decreased at the end of the eighth week.

The mean value of diametral tensile strength of (Type I) polymeric CPC significantly increased from 1 hour (4.70 MPa) up to one week (6.41 MPa) then significantly decreased at the end of the eighth week (4.58 MPa). The mean diametral strength value of (Type II) polymeric calcium phosphate cement significantly increased from 1 hour (7.39 MPa) up to one week (8.80 MPa) and slightly decreased at the end of the eighth week (8.57 MPa). The mean diametral tensile strength value of (Type III) polymeric CPC cement significantly increased from 1 hour (11.43 MPa) up to one week (14.03 MPa) then decreased at the end of the eight week (12.59 MPa).

### 3.3. Infrared Spectroscopic Analysis

**Figure 3:**
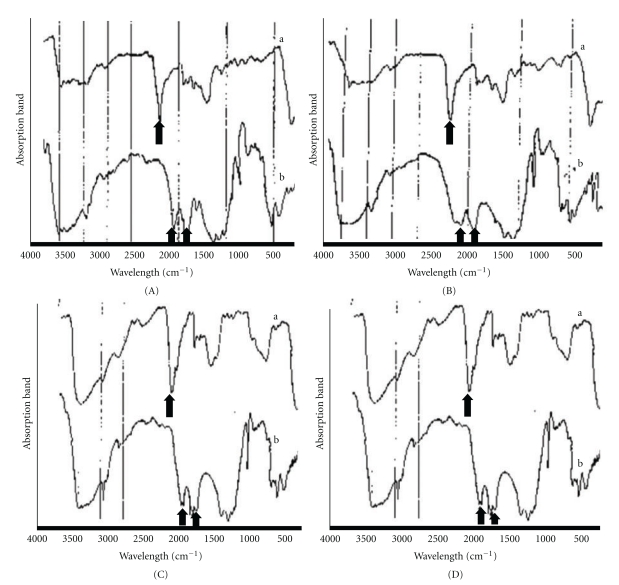
(A) Zinc polycarboxylate cement; (B) Type I; (C) Type II; (D) Type III. For all cements tested, IR spectra of polymeric acids (modified polyacrylic acid, modified polyalkenoic acid, 35% w/w aqueous solution of PMVE-Ma (a) showed the absorption bands of carboxylic group (C=O) between 1635 to 1640 cm^−1^ cm (arrows). IR spectra of set cements (b) showed the absorption bands between 1558 and 1401 cm^−1^ indicating the formation of carboxylic salts (arrows).

The infrared spectrum of modified polyacrylic acid, visible light-cure modified polyalkenoic acid, 35% (w/w) aqueous solution of polymethylvinylether-maleic acid (PMVE-Ma) and the set cements derived from their mix with CPC powder are presented in (Figures 3(A), 3(B), 3(C), and 3(D)). 

The infrared spectrum of the modified PA acid and the set zinc polycarboxylate cement (control group) (Figure 3(A)) showed a stretching band at 1638 cm^−1^, representing the (–C=O) antisymmetric stretching band of the carboxylate group in the polyacrylic acid (Figure 3(A)). The set product of zinc polycarboxylate cement (control group) showed two IR-absorption bands at 1558 and 1418 cm^−1^, (Figure 3(B)) which were assigned to a carboxylic acid salts (–COO) [27, 28].

The infrared spectrum of the modified PA acid and the set cement (Type I) (Figure 3(B)) showed a stretching band at 1638 cm^−1^ representing the carboxylic group (–C=O) of the polyacrylic acid (Figure 3(A)). The set products of modified polyacrylic acid and CPC powder (Type I) showed the disappearance of the stretching band at 1638 cm^−1^ and two other stretching bands appeared at 1558 and 1418 cm^−1^, indicating carboxylate salts formation (Figure 3(B)) [27, 28]. The infrared spectra of the light-sensitive modified polyalkenoic acid and the set cement (Type II) (Figure 3(C)) showed a stretching absorption band at 1640 cm^−1^, representing the carboxylic group (–C=O) of the modified polyalkenoic acid (Figure 3(A)). The set products of the VLC modified polyalkenoic acid and the CPC powder showed the disappearance of the stretching band at 1640 cm^−1^, and two other stretching bands appeared at 1558 and 1418 cm^−1^, indicating carboxylate salt formation (–COO) (Figure 3(B)) [27, 28]. 

The infrared spectra of the 35% w/w aqueous solution of PMVE-Ma acid and the set cement (Type III) are shown in (Figure 3(D)). The (–C=O) stretching absorption band of the carboxylic acid group of PMVE-Ma was observed at 1635 cm^−1^ (Figure 3(A)). The band at 1635 cm^−1^ disappeared in the IR spectrum of the set cement and two new stretching bands attributable to carboxylate (–COO) formation were observed at 1558 cm^−1^ and 1401 cm^−1^ indicative of acid-base reactions involving PMVE-Ma acid and CPC powder (Figure 3(B)) [19].

### 3.4. X-Ray Diffraction Analysis

**Figure 4:**
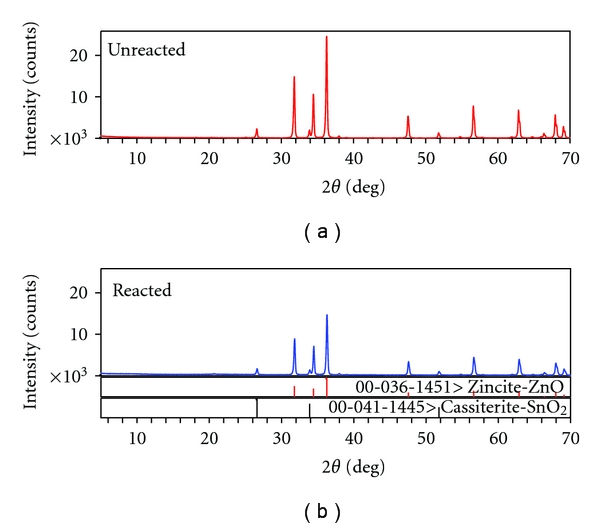
(a) X-ray diffraction pattern of unreacted zinc polycarboxylate powder. (b) X-ray diffraction pattern of set zinc polycarboxylate cement.

**Figure 5:**
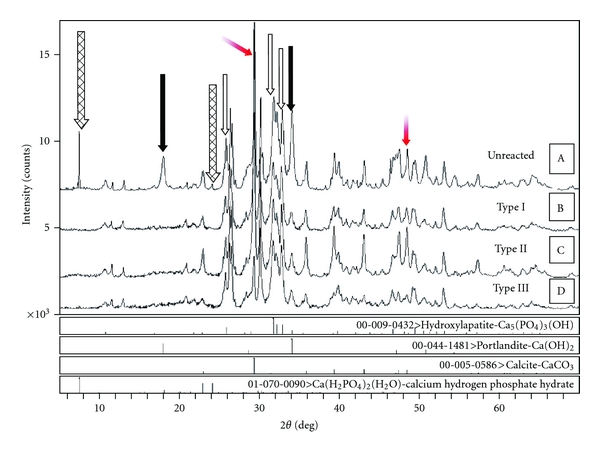
X-ray diffraction pattern of the three types of polymeric cements (Type I, Type II, and Type III CPCs). (A) Unreacted powder component. (B) Set product of Type I cement. (C) Set product of Type II cement. (D) Set product of Type III cement. Different arrows indicating the characteristic peaks of the different crystalline phases of the X-ray diffraction pattern; cross-hatched arrows indicate the crystal phase of calcium hydrogen phosphate hydrate, black arrows portlandite, grey arrows hydroxyapatite, and gradient arrows calcite.

 Two phases (zincite-ZnO and cassiterite-SnO_2_) which exhibited the characteristic peaks around 2*θ* = 36.253° and 2*θ* = 26.611°, respectively, were detected in the XRD patterns of unreacted and reacted zinc polycarboxylate cement (Figure 4). The XRD patterns for Types I, II, and III cements (Figure 5) were basically identical with four phases. The four phases and the characteristic peaks are as follows: hydroxyapatite (HA) [Ca_5 _(PO_4_)_3_(OH), 2*θ* = 31.773°], monetite [CaPO_3 _(OH), 2*θ* = 30.189°], brushite [CaPO_3 _(OH)·2H_2_O, 2*θ* = 11.681° and 20.934°], and calcite [CaCO_3_, 2*θ* = 29.400°].

### 3.5. Scanning Electron Microscopy

**Figure 6:**
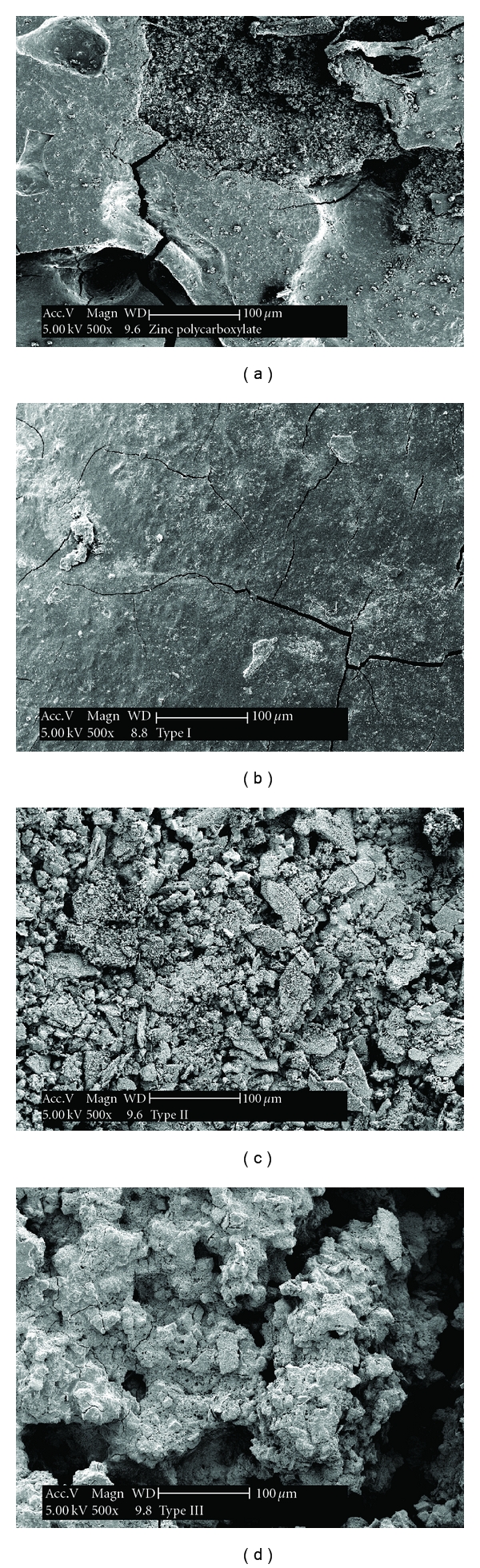
SEM microphotograph of the longitudinal top surface of the four types of cements. (a) Zinc polycarboxylate cement; (b) Type I; (c) Type II; (d) Type III after setting.

 The SEM photomicrographs in (Figures 6(a) and 6(b)) of the surface topography showed a porous surface with linear microcracks for both zinc polycarboxylate (control) and Type I CPC cements. 

**Figure 7:**
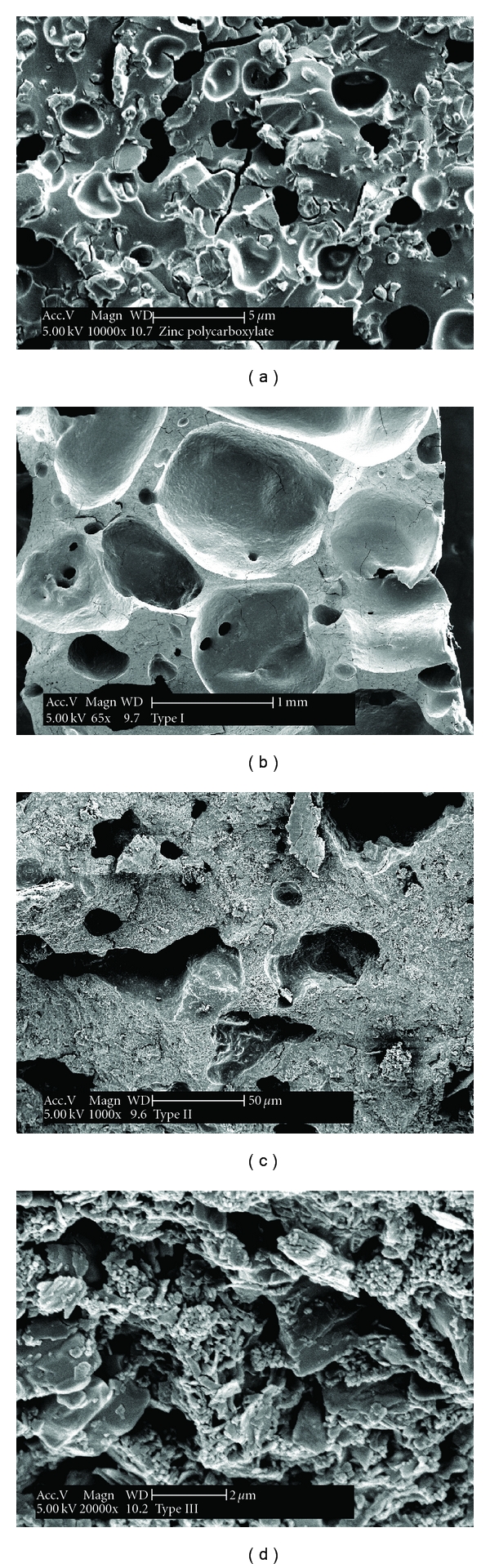
SEM microphotograph of the fractured surface of the four types of cements. (a) Zinc polycarboxylate cement; (b) Type I; (c) Type II; (d) Type III after setting.

A mixture of thin needle- or rod-shaped microcrystals characteristic of hydroxyapatite were identified on the top surface of Type II cement. These hydroxyapatite crystals were precipitated on the cement surface together with plate-like crystals as shown in (Figure 6(c)). The top surface of Type III as shown in (Figure 6(d)) exhibited flakes and sandy grain-shaped crystals. The SEM examination of the longitudinal fracture surface of zinc polycarboxylate and Type I cement showed the same porous pattern formed of small and large micropores and different sized shallow cavities (Figures 7(a) and 7(b)). Particles of Type II and Type III cements were mostly clustered into agglomerates that locally exhibited platy like crystals on their fracture surfaces (Figures 7(c) and 7(d)).

### 3.6. Cellular Mitochondrial Activity

**Figure 8:**
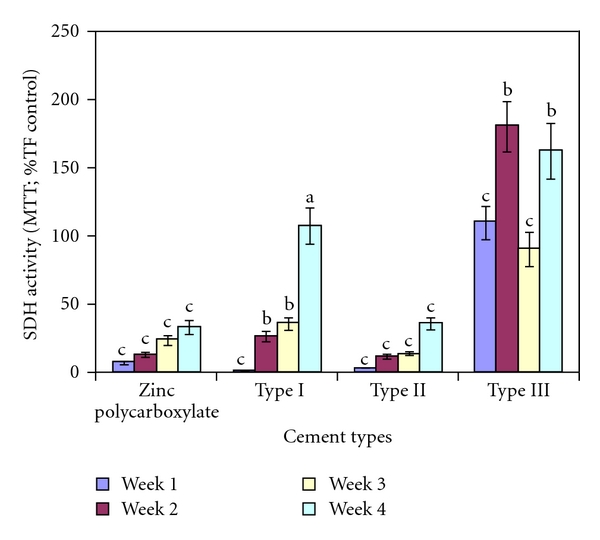
Mitochondrial suppression induced by zinc polycarboxylate, Type I, Type II, and Type III calcium phosphate cements as function of aging time. Cytotoxicity was measured by succinic dehydrogenase activity and expressed as a percentage of Teflon controls (defined as 100%). There were six replicates per condition. Different letters indicate a statistically significant difference between the materials (ANOVA, Tukey intervals *α* = 0.05).

Cellular mitochondrial suppression induced by the CPCs and zinc polycarboxylate cement (control group) is illustrated in (Figure 8). Aging influenced the mitochondrial suppression of all materials except Type II, which suppressed mitochondrial activity throughout the testing intervals. For each week, the comparison among all cement types was highly significant (*P* < 0.01). Type III cement showed an increase in SDH activity by (*>*90%) and was statistically equivalent to the negative Teflon control after 1 wk. Type III cement was significantly higher than the Teflon control at week 2 and week 4. Type I cement was severely cytotoxic (>90% suppression) relative to Teflon controls, but significantly (*P* < 0.05) improved were statistically equivalent to the Teflon control after 4 weeks. Type II cement did not change significantly in cytotoxicity over the entire four week evaluation. Type II cement was significantly (*P* > 0.05) lower than Teflon control over the entire four week evaluation period. Zinc polycarboxylate cement also significantly suppressed mitochondrial activity throughout the testing but showed some improvement by the fourth week.

## 4. Discussion

The most difficult challenge in designing and manipulating dental materials is being able to mimic the complex physical and functional characteristics of natural tissues. Development of a replacement material that either mimics natural tissue properties and performance and/or one that is eventually resorbed and replaced by equivalent new tissue is the final goal of restorative dentistry. 

Thus, hydroxyapatite (HA) materials combined with organic compounds are promising dentin replacing materials. Various calcium phosphate derivatives, for example, hydroxyapatite (HA), tricalcium phosphate (TCP), octacalcium phosphate (OCP), dicalcium phosphate (DCP), and monocalcium phosphate (MCPM) have been studied in the last decade because of their biocompatibility, osteoconductivity, and self-hardening properties which are desirable in a broad range of dental and biomedical application [17, 29–31]. The CPC cements derived from these compounds have high pH and freely available calcium ions, and both factors stimulate the precipitation of secondary dentin [32, 33]. 

In the present study, experiments have been undertaken in order to develop some nontraditional dental cementing materials. The principal compounds [synthetic hydroxyapatite (SHAp6) and calcium oxide (CaO)] that have been used, proved to have (as far as dentistry is concerned) encouraging properties which will certainly open an avenue for the material scientists to overcome some of the drawbacks encountered with well known dental cements. In the present study, all materials selected for the preparation of polymeric calcium phosphate cements: calcium oxide (CaO) monocalcium phosphate monohydrate (MCPM), and synthetic hydroxyapatite (SHAp6) powders as well as modified polymeric liquids (Polyacrylic acid “PA”), visible light cured polyalkenoic acid (VLC), and polymethyl vinyl ether maleic acid (PMVE-Ma), are all of medical grade, commercially available, and have a well-established compatibility.

Calcium oxide (CaO) is known to react rapidly with water and plays and important role in the hydration reaction of the set cement (a linear relationship was found to exist between the strength and the degree of hydration of dental cements) [34]. 

As for monocalcium phosphate monohydrate (MCPM) it is often used as the acid calcium phosphate in hydraulic calcium phosphate formulations, but commercial MCPM is not pure, contains a small amount of orthophosphoric acid and moisture, is consequently difficult to mill, and the powder is sticky and presents aggregates. 

Because granularity influences the mechanical properties of the hardened cement, it was, therefore, necessary to premix MCPM with CaO before grinding it though a rapid decrease in the amount of (MCPM) was observed during mechanical grinding by a solid-solid reaction with CaO [35]. An essential parameter was also considered before mixing these two essentials components by sieving each one of them separately up to 80 microns. The reduction of particle size was found to produce a substantial decrease of the setting time and accelerated the hardening of the cement without significantly affecting the final strength attained [13].

 Ginebra et al. (2004) stated that the cement cannot be univocally related to the degree of reaction without considering the microstructural features [36]. The sieved monocalcium phosphate (MCPM) and the calcium oxide (CaO) forming 60% by weight were then mixed at a ratio of Ca/P of 1.67 similar to that present in dentin and bone. Synthetic hydroxyapatite incorporated in the starting powder (40% by weight) is the most stable compound precipitated at the used Ca/P ratio of 1.67. Hydroxyapatite sintered at high temperature resulted in large particle size that increases the ultimate strength of the cement, which was found to be proportional to the precipitated amount of dicalcium phosphate dihydrate. When these formulated powders were mixed with water, the resultant cement had good handling characteristics but poor mechanical properties [37].

Therefore, in the present work, in order to overcome the disadvantages of mixing with water, the aqueous solutions of modified (polyacrylic acid, polyalkenoic (VLC) and polymethylvinyl ether maleic acid) were used. Their setting reaction was found to be biphasic, the first step during the mixing time, (MCPM) reacted with CaO immediately to give dicalcium phosphate dihydrate (DCPD) which in the second step, reacted more slowly with the remaining CaO to give hydroxyapatite.

An essential criterion was also considered in relation to the molecular weight and concentration of the aqueous solutions used for mixing the powder, as higher molecular weights tend to result in cements with shorter setting times and a higher compressive, diametral, and biaxial flexural strengths than lower molecular weight counter parts [37].

 The polymethyl vinyl ether maleic anhydride (PVME-Ma) is a commercial copolymer offered in several molecular weights and can be dissolved by hydrolysis of the anhydride group in water to form the corresponding maleic acid copolymer (polymethyl vinyl ether maleic acid). This copolymer has already a number of nondental applications in hair sprays and surgical adhesives, which suggests potential favorable biocompatibility for dental and other biomedical uses [20]. Because it was difficult to form workable cements from more highly concentrated solutions of PMVE-Ma due to their high viscosities, concentrations above 30% cannot be investigated. However, aqueous solutions of higher concentrations are feasible using lower molecular weight PMVE-Ma. In the present work, PMVE-Ma (50.000 molecular weight) was used in an aqueous solution of 35% w/w [19].

In order to optimize the powder liquid ratio of the mixing powder, extensive preliminary testing of various powder mixtures ratios was performed resulting in the optimal ratio of 4 : 1. 

### 4.1. Setting Time

The setting time of zinc polycarboxylate cement (control group) used in this study agreed with those values previously reported in the literature (Table 2).

The setting time results of the three formulated polymeric cements (Table 2) indicated that Type I mixed with modified polyacrylic acid showed a clinically acceptable setting time (5 min) which is mainly attributed to an initial hydration reaction between the starting cement powder and the water content of the liquid, followed by the completion stage, which is reached by the acid-base reaction and subsequent formation of carboxylate salts as confirmed in the IR spectral data of the set cement (Figure 3(B)).

As for Type II polymeric CPC cement mixed with polyalkenoic acid (VLC), the setting time (Table 2) cannot be recorded as the surface of the cement remained soft for a long time attributed to inhibited polymerization of the methacrylate group of the liquid, since no light curing was applied to primarily activate the photoinitiator in the hydroxyethylmethacrylate (HEMA) group and the presence of a residual nonfunctional carboxylic group which was observed in the IR spectra of the set cement (Figure 3(C)).

In Type III CPC mixed with (PMVE-Ma acid), a setting time of 9 min was observed. This may be attributed to the multifunctional nature of the PMVE-Ma acid which appears to react forming insoluble products which coat the cement particle. This encapsulation slightly retards their dissolution which may be attributed to residual maleic anhydride units as observed in the IR spectral data (Figure 3(D)) and subsequent conversion to less soluble hydroxyapatite [19, 20].

### 4.2. Mechanical Properties

 Results of the compressive and diametral tensile strength values of the newly formulated polymeric calcium phosphate cements (CPCs) as shown in (Tables 3 and 4) denoted outstandingly improved values when compared to zinc polycarboxylate cement (control group). These high strength values may be attributed to the synthetic hydroxyapatite which was incorporated in the starting powder. Hydroxyapatite is known to increase the strength of the cement powder and to act as a seed for nucleation of more hydroxyapatite [38]. Yang et al. reported that the seed concentration of (HA) improved crystallinity of the apatite phase, thus increasing the compressive strength [39]. 

In this study, the reaction between CPC powder and 35% (w/w) (PMVE-Ma) aqueous solution (Type III) resulted in a polymeric CPC with a compressive strength of 71.68 MPa and diametral strength of 11.43 MPa one hour after mixing. These results are in accordance with Matsuya et al. [19]. These early strength values are very beneficial and may permit their use in certain clinical applications (temporary fillings, luting cements, and endodontic sealers). At the end of the first week, maximum strength values were reached. This may be explained by the effect of the molecular weight of PMVE-Ma present in the liquid component is likely to have influenced the compressive strength by virtue of the reinforcement that can be provided by the elongated chains of the polymer matrix. The high molecular weight (50,000) of the PMVE-Ma, in addition to the highly branched structure, allows it to bridge numerous crystallites and engage in intermolecular entanglements, providing strengthening mechanisms.

The cement prepared from CPC powder and visible light cure polyalkenoic acid (VLC) Type II had a compressive strength value of 66.86 MPa and diametral tensile strength of 7.39 MPa, 1 hr after mixing and reached its maximum value at the end of 24 hours. These results coincide with Miyazaki et al. [28]. The results were confirmed by the IR analysis of the reaction products, which showed that the reaction has been completed as evident by the disappearance of the stretching band (C=O) carboxylic group and the formation of carboxylic salts. The compressive and tensile strength of Type II and Type III polymeric CPC were significantly higher than that of zinc polycarboxylate (control group).

Storage in distilled water at 37°C slightly affected the mechanical properties, therefore suggesting a stable formula that can resist disintegration in the oral environment.

### 4.3. Setting Reactions

Multifunctional acids such as polymethylvinyl ether maleic acid (PMVE-Ma) and polyacrylic acid (PA) are characterized by the presence of carboxylic groups [21]. The calcium ions released from CPC powder (MCPM and CaO) mainly react with the carboxylic group of the polyacids and become cross-linked to the polyacid chains by an ion exchange or an acid base reaction [20, 28]. This setting mechanism was previously described in other types of polymeric calcium phosphate cements [27]. The amorphous reaction products (polysalts) derived from the reaction of PMVE-Ma acid and PA acid with the CPC powder form a cement matrix analogous to that formed in zinc polycarboxylate and glass ionomer cement [20, 40–42]. 

The use of total reflectance of infrared spectroscopy makes it possible to monitor the setting reaction and the transformation of the COOH group to COO groups. 

The infrared spectra of the set polymeric CPC Types (I, II, and III) 24 hours after mixing showed the absence of the stretching peak of carboxylic group (–COOH) and the appearance of two new carboxylate stretching peaks indicating the formation of the polyacrylic salts (Figures 3(A), 3(B), 3(C), and 3(D)).

### 4.4. X-Ray Diffraction

The X-ray diffraction analysis of zinc polycarboxylate unreacted powder identified two crystalline phases ZnO and SnO_2_. The presence of tin oxide depends on the commercial cement initially used: it is present in small quantities in some cements and not in others. No zinc polycarboxylate reflections were seen, since this compound is amorphous. Therefore, the cement must be a composite of unreacted oxides ZnO (mainly) and SnO_2_ and the amorphous zinc polycarboxylate matrix resulting from the setting reaction of these oxides with polyacrylic acid and water. These results are in accordance with the core link structure proposed for such cements [43]. The X-ray diffraction analysis of the unreacted CPC powder identified four phases: hydroxyapatite [Ca_5 _(PO_4_)_3_(OH)], monocalcium phosphate monobasic hydrate [Ca(H_2_PO_4_)_2_·H_2_O], calcite [CaCO_3_], and portlandite [Ca(OH)_2_].

During the hydration of cements consisting of nominally 60wt % monocalcium phosphate monobasic and calcium oxide and 40 wt% hydroxyapatite, two dibasic phosphate compounds, (monetite and its dihydrate, brushite) were identified in the sample. Monocalcium phosphate monobasic (MCPM) reacted with excess water forming monetite and/or brushite by

(1) $$\begin{array}{cc} & \text{Ca}{\left({\text{H}}_{2}\;\text{PO}\right)}_{2}\cdot {\text{H}}_{2}\text{O}\;+{\text{H}}_{2}\text{O}\to {\text{CaHPO}}_{4}\;\left(\text{monetite}\right)\\ & \;+{\text{H}}_{3}{\text{PO}}_{4}+2{\text{H}}_{2}\text{O}\to \text{Ca}\;{\text{HPO}}_{4}\cdot 2{\text{H}}_{2}\text{O}\;\left(\text{brushite}\right). \end{array}$$

Calcium oxide is very hygroscopic and readily converts to hydroxide by reacting with water vapor in air. Portlandite in turn absorbs CO_2_ from air forming calcite.

### 4.5. Cellular Mitochondrial Activity

Although mechanical and physical properties are of great concern for dentin regenerating pulp capping, lining, or base material, biocompatibility is another critical issue. The current study established that 4 weeks aging of the developed formulations of calcium phosphate cements may significantly change their ability to alter cellular function. However, the effect was not uniform for all formulations. Type I calcium phosphate cement (CPC mixed with polyacrylic acid) showed less mitochondrial suppression with time. It is possible as cytotoxic elements leached from the material, they either complexed with other molecules in the medium or broke down into smaller components, in each case rendering it less cytotoxic. 

 For Type II calcium phosphate cements mixed with resin modified glass ionomer (Vitremer), suppressed cellular activity was ongoing, suggesting that leaching of components with biological liabilities remained even after 4 weeks of aging. The Vitremer liquid is a light sensitive, aqueous solution of a modified polyalkenoic acid and contains 2 hydroxy ethyl methacrylate (HEMA). In most dental resin modified glass ionomer cements, HEMA is often used as a comonomer to render the resin modified polyacid compatible with water [44, 45]. However, HEMA was believed to cause potential cytotoxicity to the surrounding tissue if not completely polymerized. This may have led to a greater change in the pH of the culture medium, resulting in more cellular damage and represented by the suppression of mitochondrial activity. Type III CPC mixed with aqueous solution of polymethyl vinyl ether maleic acid, however, exhibited compatibility equivalent to Teflon over the entire time period with the exception of weeks 3 and 4 time points, where it is several folds higher than Teflon. A stimulation of the metabolic activity of the cells in culture by the Type III CPC after 4 weeks is not easily explained and warrants further investigation. Polymethyl vinyl ether malice anhydride (PMVE-Ma) is a commercial copolymer offered in several molecular weights and can be dissolved by hydrolysis of the anhydride group in water to form the corresponding maleic copolymer (poly methyl vinyl ether maleic acid). This copolymer has already a number of nondental applications including hair sprays, and surgical adhesives, which suggests potential for favorable biocompatibility for dental and other biomedical uses [19]. 

 We prepared the CPCs by mixing calcium oxide, calcium phosphate monohydrate (MCPM), and synthetic hydroxyapatite. Calcium oxide (CaO) is known to react rapidly with water and plays an important role in the strength and degree of hydration of dental cements. As for monocalcium phosphate monohydrate (MCPM), it is often used as the acid-calcium in hydraulic calcium phosphate formulations. Calcium phosphate biomaterials are thought to generally be biologically well tolerated, because the main inorganic constituents of bone, hydroxyapatite is comprised of calcium and phosphate [46, 47]. 

Interestingly, the cytotoxicity of the three different formulations is dependent on the composition of the polymeric acid used for mixing. In our experimental design, cytotoxicity was estimated by mitochondrial succinate dehydrogenase activity (SDH) activity in the MTT assay and expressed as a percentage of the Teflon negative control value (100%) being equivalent to Teflon with no evident cytotoxicity. 

On the other hand, the biocompatibility of zinc polycarboxylate cement (control group) has also been investigated. Our data show that zinc polycarboxylate cement was the most cytotoxic of the tested materials in accordance with previous data [48, 49].

## 5. Conclusion

Type III CPC presented reasonable setting time, significantly higher compressive, and diametral tensile strengths when compared to zinc polycarboxylate cement (control group).

By virtue of these characteristics coupled with its biocompatibility, Type III CPC cement shows promise for dental applications.

## Figures and Tables

**Table 1:** Materials used in this study and their manufacturers.

Material	Composition	Trade Name	Manufacturers
(1) Zinc polycarboxylate cement	Powder: Zinc oxide with traces of Mg oxide and Sn oxide. Liquid: aqueous solution of polyacrylic acid and in taconic acid.	G.C.R.	Advanced Research IncDental division England
(2) Glass Ionomer liquid light cured modified polyalkenoic acid	A light sensitive aqueous solution of polyalkenoic acid modified with methacrylic group.	Vitremer	3M Dental products St. Louis, USA
(3) Monocalcium phosphate monobasic (MCPM)		Calcium Phosphate Monobasic	Sigma Chemical Co., Aldrich GmbhGermany
(4) Calcium oxide (CaO)			Adwic Laboratory Chemical
(5) Polymethyl vinyl ether maleic anhydrate copolymer (white powder) PMVE-Ma			Sigma-chemical Laboratories St. Louis, USA
(6) Synthetic hydroxylapatite (SHAP_6_)			Prepared at the Department of Dental Materials of the Medical College of Georgia, Augusta, Ga, USA

**Table 2:** The initial setting time (in minutes) of zinc polycarboxylate cement and the three polymeric calcium phosphate cements (CPCs).

	Zinc polycarboxylate (control group)	Polymeric calcium phosphate			L.S.D.5%
		Type I	Type II	Type II	
Mean setting time (in minutes) ± SD	5 ± 1	5 ± 1	—	9 ± 1	1.30*

NB: Type II polymeric CPC (no setting reaction) VLC type.

*Significant at 5% level.

**Table 3:** Mean compressive strength and standard deviation of zinc polycarboxylate cement and the three polymeric calcium phosphate cements (CPCs) in MPa.

Cement types		1 hour	24 hours	1 week	4 weeks	8 weeks	*F*-value
Control zinc polycarboxylate		46.85 ± 3.51	50.83 ± 4.50	50.87 ± 2.65	51.88 ± 2.80	52.60 ± 2.95	4.33*
Calcium phosphate cements (CPCs)	Type I	40.42 ± 3.33	44.87 ± 3.25	49.80 ± 2.75	48.74 ± 2.80	46.91 ± 3.65	3.90*
	Type II	66.86 ± 1.38	67.13 ± 1.30	67.15 ± 1.38	67.20 ± 1.30	67.12 ± 1.22	1.74*
	Type III	71.68 ± 3.15	75.12 ± 3.55	75.56 ± 2.75	73.62 ± 2.95	73.59 ± 3.10	4.09*
	LSD 5%	3.81*	4.22*	3.19*	3.30*	3.32*	

*Significant at 5% level.

**Table 4:** Mean diametral tensile strength and standard deviation of zinc polycarboxylate cement and the three polymeric calcium phosphate cements (CPCs) in MPa.

Cement types		1 hour	24 hours	1 week	4 weeks	8 weeks	*F*-value
Control zinc polycarboxylate		5.65 ± 1.91	5.92 ± 2.35	4.49 ± 1.91	5.50 ± 1.99	4.80 ± 1.51	2.55*
Calcium phosphate cements (CPCs)	Type I	4.70 ± 1.84	5.85 ± 2.41	6.41 ± 2.04	5.55 ± 2.36	4.58 ± 1.84	2.77*
	Type II	7.39 ± 1.99	8.74 ± 1.74	8.80 ± 2.02	8.63 ± 2.51	8.57 ± 2.96	2.96*
	Type III	11.43 ± 2.37	13.81 ± 2.22	14.03 ± 1.95	12.77 ± 1.89	12.59 ± 1.57	3.66*

*Significant at 5 % level.
